# Global Vision of the Reaction and Deactivation Routes in the Ethanol Steam Reforming on a Catalyst Derived from a Ni–Al Spinel

**DOI:** 10.1021/acs.energyfuels.4c00646

**Published:** 2024-04-09

**Authors:** Sergio Iglesias-Vázquez, José Valecillos, Aingeru Remiro, Beatriz Valle, Javier Bilbao, Ana G. Gayubo

**Affiliations:** Department of Chemical Engineering, University of the Basque Country (UPV/EHU), P.O. Box 644, Bilbao 48080, Spain

## Abstract

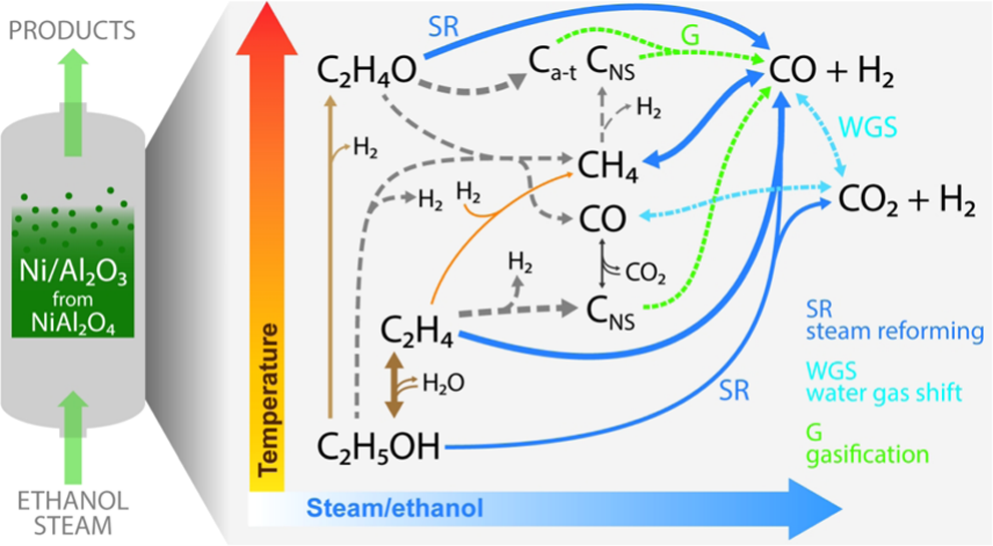

Ethanol steam reforming (ESR) over a Ni/Al_2_O_3_ catalyst prepared by reduction of a NiAl_2_O_4_ spinel is a promising alternative route to produce H_2_ from biomass. This work deepens into the effect of reaction conditions (450–650 °C, a steam/ethanol (S/E) ratio of 3–9, and a weight space time up to 1.3 h) and evaluates the time on stream evolution of the yields of H_2_, gaseous byproducts (CO, CO_2_, CH_4_, C_2_H_4_, C_2_H_4_O), and formed carbon/coke. The results are explained taking into consideration the thermodynamics, the extent of each individual reaction, and the catalyst deactivation. Up to 600 °C, the predominant intermediate in the H_2_ formation is C_2_H_4_ (formed by ethanol dehydration) with the preferential formation of nanostructured carbon (nanotubes/filaments) by C_2_H_4_ decomposition. The deposition of this type of carbon partially deactivates the catalyst, mainly affecting the extent of the C_2_H_4_ decomposition causing a sharp decrease in the H_2_ and carbon yields. Nevertheless, the catalyst reaches a pseudosteady state with an apparent constant activity for other reactions in the kinetic scheme. At 650 °C, C_2_H_4_O (formed by the ethanol dehydrogenation) is the main intermediate in the H_2_ formation, which is the precursor of an amorphous/turbostratic carbon (coke) formation that initially causes a rapid deactivation of the catalyst, affecting the ethanol dehydration and, to a lower extent, the reforming and water gas shift reactions. The increase in the S/E ratio favors the H_2_ formation, attenuates the catalyst deactivation due to the suppression of the ethanol dehydration to C_2_H_4_, and promotes the reforming, water gas shift, and carbon/coke gasification reactions. A H_2_ yield of 85% stable for 48 h on stream is achieved at 600 °C, with a space time of 0.1 h and an S/E ratio of 9.

## 1 Introduction

H_2_ is a key raw material in the chemical industry for ammonia synthesis, hydroprocessing of petroleum fractions, Fischer–Tropsch, fuel synthesis, and many other hydrogenation reactions. Additionally, it is considered an ideal energy carrier to satisfy the growing demand for energy due to its high energy density (143 kJ kg^–1^). In this context, its combustion causes no carbonaceous emissions (only H_2_O as a product), which makes it a promising solution to abate the problems arising from the emissions of greenhouse gases and other contaminants coming from fossil resources.

The development of the H_2_ economy is based on four cornerstones (production, storage, transportation, and use).^1^ Nowadays, more than 90% of the H_2_ production (estimated to be 120 million tons by 2024)^2^ is carried out by the catalytic steam reforming (SR) of fossil feedstock (methane, natural gas, naphtha, propane), with high emissions of greenhouse gases (110 g CO_2_e per MJ of H_2_ from methane).^3^ To avoid the impact of these emissions on the climate change, the development of H_2_ production technologies from biomass feedstock is experiencing a growing interest in the transition scenario toward the H_2_ production through water electrolysis. These technologies include the sequential pyrolysis-reforming of biomass in tandem reactors^4^ and the reforming of biomass derivatives, such as bio-oil (product of fast pyrolysis of biomass),^5,6^ methanol (obtained from biomass gasification),^7^ and ethanol (bioethanol from biomass fermentation).^8^ It is remarkable the bioethanol availability, whose production is predicted to expand up to 80 billion gallons by 2050 due to the increasing valorization of lignocellulosic, agricultural, and forestry wastes into ethanol.^9^

The H_2_ production from bioethanol avoids the costly separation and purification steps to remove H_2_O that are traditionally required for the use of ethanol as a fuel, and it can be carried out by means of different technologies:^10^ steam reforming (SR), partial oxidation (PO), autothermal reforming (ATR), and dry reforming (DR). The know-how of the industrially extended SR of methane/natural gas provides the fundamentals (catalysts, reaction mechanisms, and reactor design) for the prospective scale-up of the ethanol steam reforming (ESR) process in the short term. Likewise, the development of H_2_ purification operations has also reached a high technological level in the SR of methane/natural gas, comprising membrane separation and reactors to convert the residual CO by water gas shift (WGS) or selective oxidation reactions.^9,11^

The reactions involved in the ESR are summarized in Table 1, including reactions well established in the literature^12−15^ for the formation of the desired products (H_2_ or syngas), byproducts (CO, CO_2_, and CH_4_), and also the formation of solid carbon materials. The latter may have a key role in catalyst deactivation depending on its origin and nature. The carbon formed by the decomposition of C_2_H_4_ (eq 11), CH_4_ (eq 12), and CO disproportionation (eq 13, also known as the Boudouard reaction) is generally filamentous or nanostructured, whose nature has poor incidence in the catalyst deactivation.^16^ Conversely, the carbon (coke) formed from the degradation of C_2_H_4_O (eq 15) is generally amorphous or turbostratic being able to encapsulate the active sites and therefore precipitating the catalyst deactivation.^17,18^ The carbon/coke formed from the C_2_H_4_ oligomerization, cyclization + dehydrogenation, and condensation (eq 14) on the acid sites is also amorphous and has a high deactivating effect. The gasification of the different carbon/coke types (eq 16) contributes to limiting their evolution.

**Table 1:** Main Reactions Involved in the ESR Process

name	chemical equation
global ethanol steam reforming	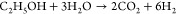 1
ethanol steam reforming	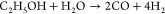 2
water gas shift (WGS)	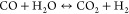 3
ethanol dehydration	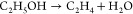 4
ethanol dehydrogenation	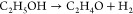 5
decomposition to gases	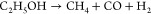 6
	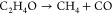 7
steam reforming (SR)	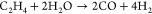 8
	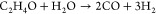 9
	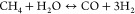 10
carbon formation	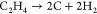 11
	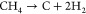 12
	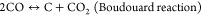 13
	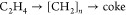 14
	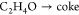 15
carbon gasification	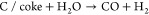 16

The catalyst and reaction conditions are the main factors that tip the balance toward certain products by favoring selectively the extent of reactions in Table 1. The most common catalysts for the ESR are based on Ni or Co supported on various oxides and less commonly based on noble metals, such as Pt or Rh.^8−10,19,20^ Those based on Ni are preferred due to their high activity in the steps of the reforming reaction mechanism, including C–C and C–H bond cleavage, H_2_O adsorption and dissociation, and their comparatively low cost.^21,22^ The problem of rapid deactivation of the Ni catalysts by sintering and coke deposition has been a topic of interest to study different preparation strategies and formulations.^15,23,24^ The dehydrogenation reactions are favored on noble metals, which leave dehydrogenated surface species that are effectively oxidized to CO or CO_2_, suppressing the formation of CH_4_ and carbon.^25^ Regarding the important role of the catalyst support, the properties that determine the reaction routes are its acidity/basicity/neutrality, hydrophilicity, and oxygen mobility capacity. Acidic supports (e.g., acidic Al_2_O_3_) promote ethanol dehydration, producing C_2_H_4_ as an intermediate,^26−28^ whereas other supports (e.g., SiO_2_ or neutral La_2_O_3_–Al_2_O_3_) can promote ethanol decomposition to CH_4_ and CO, or dehydrogenation, producing C_2_H_4_O as an intermediate.^13,17,29−31^ On the other hand, hydrophilic supports (e.g., ZrO_2_), and those with high oxygen mobility capacity (due to their oxygen vacancies, e.g., CeO_2_), facilitate the H_2_O-consuming reactions by contributing to the formation of OH species and to oxidative reactions.^9,22,25^

Reaction conditions (such as temperature, steam/ethanol (S/E) ratio, and contact time) also strongly affect the extent of the reactions in Table 1, some of them being limited by the thermodynamic equilibrium.^32−34^ Increasing the reaction temperature would favor the SR, decomposition, and gasification reactions and would disfavor the extent of the WGS (moderately exothermic) and Boudouard reactions. Increasing the S/E ratio would benefit all of the reactions that consume H_2_O, including SR, WGS, and gasification reactions. Increasing the contact time, frequently defined as the ratio between the catalyst mass and feed flow rate, would expectedly favor the extent of all of the catalytic reactions, with a major influence on the SR and WGS reactions. However, the carbon/coke formation by thermal or catalytic routes may deactivate the catalyst, decreasing the extent of all of the catalytic reactions over time on stream and therefore modifying the product distribution.^10^ Although the main focus when designing a catalyst for the ESR is to improve H_2_ production while avoiding carbon/coke formation,^14,35^ catalysts based on Ni supported on Al_2_O_3_ or CaO have been successfully used, at particular conditions, for the coproduction of H_2_ and nanostructured carbon (filamentous/nanotubes). These carbon materials are valuable products for many applications in electronics, biomedicine, catalysis, and hydrogen storage.^36−39^ He et al.^40^ prepared carbon nanotubes from steam reforming of pyrrole over Ni–Fe/Ni foam catalysts with excellent electrochemical performance for their use as supercapacitors.

The reduction of a NiAl_2_O_4_ spinel synthesized by coprecipitation involves the exsolution of Ni from the spinel structure, forming reduced Ni crystals deposited on Al_2_O_3_. We previously demonstrated that the resulting Ni/Al_2_O_3_ catalyst has smaller and more uniform Ni crystals and a more homogeneous distribution in the Al_2_O_3_ support than that prepared by impregnation with a similar Ni content, which is advantageous to achieve higher H_2_ yields.^41^ Other advantages of using this catalyst are the simple and reproducible preparation, resistance to sintering,^42,43^ and regeneration capacity (by coke combustion, with simultaneous reconstruction of a NiAl_2_O_4_ spinel).^44,45^ In addition, this catalyst offers flexibility/adaptability to different operating strategies aimed at producing H_2_ and carbon filamentous/nanotubes, the latter produced by decomposition of the intermediate compound C_2_H_4_, whose formation is promoted by the acidity of the Al_2_O_3_ support.^28^

In this work, we have carried out a more detailed study of the effect of the reaction conditions (temperature, space time, S/E ratio) on the performance of the ESR reaction on the catalyst derived from a NiAl_2_O_4_ spinel, considering the thermodynamics, extent of each reaction (Table 1), and the catalyst deactivation. The results provide an overview about the capacity of this catalyst and allow the establishment of adequate reaction conditions to maximize the H_2_ yield while minimizing catalyst deactivation by coke (prolonging the catalyst lifetime prior to be regenerated). Additionally, the knowledge of the relationship between the extent of the reaction routes in the ESR and the reaction conditions will be useful to progress toward the development of a rigorous kinetic model for this process considering the catalyst deactivation.

## 2 Experimental Section

### 2.1 Catalyst Preparation and Characterization

The catalyst was prepared from a NiAl_2_O_4_ spinel precursor following the procedure described in previous work.^28,42,44^ The NiAl_2_O_4_ spinel was synthesized by coprecipitating Ni(NO_3_)_2_ and Al(NO_3_)_3_ while dosing a NH_4_OH solution dropwise to reach a pH of 8. The precipitate was calcined at 850 °C for 4 h in a static air atmosphere to obtain the NiAl_2_O_4_ spinel and then crushed and sieved at 0.15–0.25 mm. The catalyst was obtained by reduction of the NiAl_2_O_4_ spinel at 850 °C for 4 h with 10 mol % H_2_ in N_2_ and a heating rate of 10 °C min^–1^ in the reaction system.

The NiAl_2_O_4_ spinel and catalyst were characterized using X-ray diffraction (XRD), temperature programmed reduction (TPR), N_2_ physisorption, and NH_3_ adsorption, whose experimental procedures and results were previously described.^28^ The structural phases detected by XRD were identified using the database of the International Center for Diffraction Data by matching with the appropriate Powder Diffraction File version 4 (PDF-4). Briefly, the characterization results confirm that^28^ (i) the NiAl_2_O_4_ spinel structure was obtained upon the precipitate calcination at 850 °C; (ii) the Ni species in the NiAl_2_O_4_ spinel were reduced above 800 °C; and (iii) the reduction treatment at 850 °C for 4 h led to reduced Ni crystals supported on Al_2_O_3_ with an acidity of 0.038 mmol g^–1^ (based on NH_3_ adsorption).

### 2.2 Catalytic Runs

The ESR reaction runs were carried out in the reaction system (microactivity reference-PID Eng & Tech) described in a previous work.^28^ Briefly, the setup is provided with an isothermal fluidized bed reactor (22 mm internal diameter and total length of 460 mm) inside a furnace, and this arrangement (reactor and furnace) is inside a hotbox kept at 150 °C. The catalytic bed in the reactor consists of a mixture of an inert material (SiC from VWR Chemicals sieved at 105 μm) and the catalyst, keeping an initial bed height/diameter ratio above 2 for all of the experiments. The feed system consists of lines of various gas streams (N_2_ used as the diluent and H_2_ used for the reduction of the spinel), each one controlled with mass flow meters, and a liquid stream (ethanol–water mixture) provided with a piston pump (Gilson 307). The mixing of the different feed components (gas or liquid streams) takes place in the hotbox kept at 150 °C to allow the evaporation of liquid components and preheating of the feed. The outlet stream from the reactor is sampled through a capillary, and the rest of the flow goes to a separator with a Peltier cooler, where the vapor components are condensed and collected, while the gas components are safely vented. The sample is mixed and carried with He to an Agilent 3000 micro gas chromatograph (micro-GC) through a thermally insulated line for the component analysis. The micro-GC has four column modules for the detection and quantification of the reaction components: (1) molecular sieve capillary column for separating O_2_, N_2_, H_2_, CO, and CH_4_; (2) PLOT Q capillary column for separating hydrocarbons (C_1_–C_3_), CO_2_, and water; (3) alumina capillary column for separating C_2_–C_4_ hydrocarbons; and (4) Stabilwax type column for separating oxygenates (C_2+_) and water. After integration of the chromatograph data and using the calibration factors, the ethanol conversion (*X*) and product yields (*Y*_*i*_) are calculated as follows
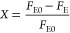
17
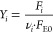
18where *F*_E0_ is the ethanol flow rate at the reactor inlet, *F*_E_ is the ethanol flow rate at the reactor outlet, *F*_*i*_ is the product *i* flow rate at the reactor outlet, and ν_*i*_ is the stoichiometric coefficient for the product *i*, which is 6 (for H_2_), 2 (for CO, CO_2_, and CH_4_), and 1 (for C_2_H_4_ and C_2_H_4_O). Consequently, the values of the conversion and yield of products shown in the following sections are dimensionless. The ESR reaction runs were carried out at atmospheric pressure, ethanol partial pressure (*P*_E0_) of 0.05 bar, and varying one at a time the reaction conditions in the following ranges: 450–650 °C; S/E molar ratio, 3–9; weight space time, 0 (no catalyst, thermal reaction), 0.01–0.2 h.

### 2.3 Thermodynamic Equilibrium Predictions

The distribution of components in thermodynamic equilibrium was predicted by the Gibbs energy minimization by using a Gibbs reactor in the commercial AVEVA PROII simulation software, as described in previous work.^5,46^ The components considered were N_2_, H_2_O, ethanol (C_2_H_5_OH), H_2_, CO, CO_2_, CH_4_, C_2_H_4_, acetaldehyde (C_2_H_4_O), and carbon (graphite), and the thermodynamic method used was Soave–Redlich–Kwong (SRK). The calculation procedure was validated in a previous work by comparing the results obtained with SRK and Peng–Robinson models, as well as the PROII software against other commercial software (DWSim 6.4.3).^5^ The results showed that the differences in the molar rates of the products calculated with SRK and PR models were insignificant (less than 0.05% relative error), and the percentage relative error in the product molar rates calculated with both simulation software was below 3.5%.

The inlet conditions in the thermodynamic calculations were similar to those used for the reaction runs considering three feed compositions (molar percentages of ethanol/H_2_O/N_2_): 5/15/80, 5/30/65, and 5/45/50. A case study was defined to predict the effect of the reaction temperature on the equilibrium composition of the components for each feed composition. Accordingly, the temperature in the Gibbs reactor was varied from 400 to 900 °C and the molar flow rates of the components at the outlet were registered for each temperature. The molar fraction and yields of the compounds were calculated using the predicted molar flow rates.

## 3 Results

### 3.1 Effect of Reaction Conditions at Zero Time: Equilibrium Approaches

This section evaluates the role of the catalyst in the ESR process as well as the interest of thermodynamic study in predicting the effect of the reaction conditions on the product distribution. As described in the Supporting Information (Figure S1), the ethanol conversion barely proceeds without a catalyst, and the main reactions taking place are the ethanol dehydration (eq 4) and dehydrogenation (eq 5) and acetaldehyde decomposition (eq 7), whereas SR reactions seem to be negligible at these conditions (as evidenced by the almost equal amounts of CO and CH_4_).

**Figure 1:**
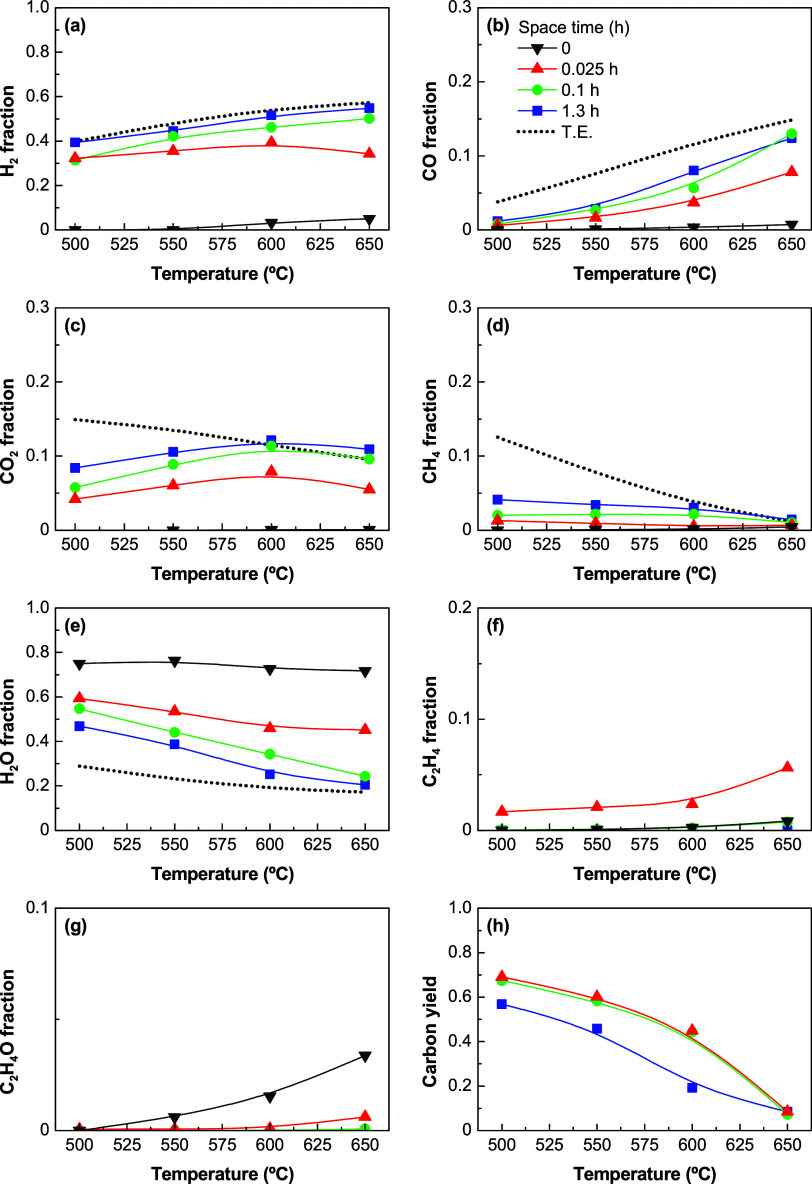
Effect of space time on the product distribution (N_2_ free molar fraction on a wet basis) at different temperatures over the Ni/Al_2_O_3_ catalyst derived from the NiAl_2_O_4_ spinel (solid lines) and comparison with the thermodynamic equilibrium predictions (dashed lines): (a) H_2_, (b) CO, (c) CO_2_, (d) CH_4_, (e) H_2_O, (f) C_2_H_4_, and (g) C_2_H_4_O. The yield of (h) carbon is calculated by C atom balance. Reaction conditions: S/E ratio, 3.

Figure 1 shows the molar fractions of components as a function of the temperature at zero time on stream with various catalyst loads (weight space times of 0.025, 0.1, and 1.3 h) and for an S/E ratio of 3. The experimental data (solid lines) are compared with the thermal reaction (space time of zero, without catalyst, black solid lines) and thermodynamic equilibrium predictions (dashed lines) at the same reaction conditions. Likewise, Figure 1h shows the yield of carbon formed as a solid product, which was determined indirectly by the C atom balance of the gaseous products and the ethanol fed. Based on the experimental results, the use of the catalyst fully converts ethanol at all of the temperature values tested (the ethanol molar fraction is zero) in comparison with the thermal reaction, bringing a significant increase in the fractions of H_2_, CO, CO_2_, and CH_4_ as the main products. The H_2_ concentration (Figure 1a) increases very rapidly for low values of space time, and then it remains almost constant for space time above 0.1 h, with values that increase steadily with increasing temperature values. The CO concentration (Figure 1b) increases with both space time and temperature, and the CO_2_ concentration (Figure 1c) also increases with space time, but it reaches an apparent maximum at 600 °C at all of the space time values. The CH_4_ concentration (Figure 1d) increases with an increasing space time and decreases with an increasing temperature. The H_2_O fraction (Figure 1e) decreases with increasing space time and temperature, which may be indicative of its consumption in some reactions and its disfavored formation in other reactions. C_2_H_4_ (Figure 1f) and C_2_H_4_O (Figure 1g) are barely detected at a low space time (0.025 h) or in the reactions without catalyst (being more detectable at high-temperature values), which indicates that these intermediates are fully converted with enough catalyst (high space time values). Apart from these gaseous components, the carbon formation is noteworthy in all of the reaction runs using a catalyst. Figure 1h reveals an extraordinary carbon yield between 500 and 600 °C, which decreases with increasing temperature and space time (more pronounced as the space time increases from 0.1 to 1.3 h). Although the increase in the space time from 0.1 to 1.3 h was intended to approach the equilibrium data, the results evidence that this increase does not significantly increase the fractions of H_2_ and CO but increases those of CO_2_ and CH_4_ while decreasing the carbon yield.

Taking into consideration the reactions in the ESR process (Table 1), the results in Figure 1 indicate that the presence of a catalyst favors the SR (eqs 1, 2, and 8–10), WGS (eq 3), and methanation (reverse of eq 10) reactions. At higher temperature values, the SR reactions are favored and the WGS and methanation reactions are disfavored, as it is well established in the literature.^17,29−32^ The carbon formation on the catalyst may occur through different mechanisms, including (i) ethanol dehydration (eq 4) catalyzed on the Al_2_O_3_ acid sites and subsequent C_2_H_4_ decomposition (eq 11) on Ni sites;^28^ (ii) the CH_4_ decomposition (eq 12), (iii) the Boudouard reaction (eq 13); (iv) C_2_H_4_ oligomerization, aromatization, and condensation into polycyclic aromatic coke (eq 14); and (v) the C_2_H_4_O degradation to coke (eq 15). The mechanisms (ii) and (iii) take place on Ni sites, whereas the mechanisms (iv) and (v) take place on acid sites. When the catalyst load is increased about 13 times (at a space time of 1.3 h), the carbon formation reactions are disfavored over other reactions, in particular, the CO conversion by the WGS reaction to give more CO_2_ and H_2_ and the CO conversion by the methanation reaction below 600 °C (consuming H_2_), which explains the almost constant H_2_ formation above 0.1 h space time.

When compared with the thermodynamic equilibrium predictions, there is an evident difference between the predicted values and the experimental data, and these differences decrease with the increase in the space time. The major differences are for the fractions of CO_2_, CH_4_, and H_2_O (Figure 1c–e, respectively) at low-temperature values (500–550 °C), whereas the differences are much lower for all of the molar fractions above 600 °C. However, the null concentrations of C_2_H_4_ and C_2_H_4_O predicted thermodynamically are observed experimentally at high space time values. These differences between the thermodynamic predictions and the experimental data are attributable to the formation of filamentous carbon, which is an unexpected component in thermodynamic equilibrium at these conditions. However, the consideration of other carbon allotropes (like filaments or nanotubes) in the thermodynamic calculations may reduce the difference between the thermodynamic predictions and the experimental data,^34^ but only carbon graphite is available in the database of many commercial simulation software. The main filamentous carbon formation reactions in Table 1 (from C_2_H_4_, and CH_4_ decompositions (eqs 11 and 12, respectively) and Boudouard reaction (eq 13)) compete with the expected reactions for the formation of gaseous products (SR, decomposition, and WGS), altering the distributions of H_2_, CO, CO_2_, and CH_4_. It should be noted that the formation of amorphous/turbostratic coke from C_2_H_4_ or C_2_H_4_O (eqs 14 and 15, respectively) would not significantly change the yield of carbonaceous gas products due to the low content of coke formed by these routes.^29^ The carbon/coke formation is directly attenuated by the gasification reaction (eq 16), whose extent is favored as the temperature is increased.

The increase in the S/E ratio is expected to promote the equilibrium approach experimentally since it favors the extent of the reactions consuming H_2_O (WGS (eq 3), SR (eqs 1, 2, and 8–10), and carbon gasification (eq 16)).^29^ This effect is observed in Figure S2 when comparing the experimental data (solid lines) with the corresponding thermodynamic equilibrium predictions (dashed lines). The C_2_H_4_ and C_2_H_4_O concentrations are not represented in this figure because these intermediates were not detected in these reactions with a high space time.

### 3.2 Effect of Reaction Conditions on Catalyst Deactivation

This section shows the effect of temperature, space time, and S/E molar ratio in the feed on the evolution of ethanol conversion and product distribution (expressed in terms of product yields) with the time on stream. The ranges of the reaction conditions studied have been defined considering the results of Section 3.1. It should be noted that the deactivation of the catalyst will be explained only based on the differences in carbon/coke deposition under different operating conditions, ruling out the deactivation caused by sintering as proven in previous work.^42,43^ This catalyst has a high resistance to sintering due to the severe conditions of preparation (both calcination of the precursor (NiAl_2_O_4_) and reduction at 850 °C to obtain the catalyst).

#### 3.2.1 Temperature

**Figure 2:**
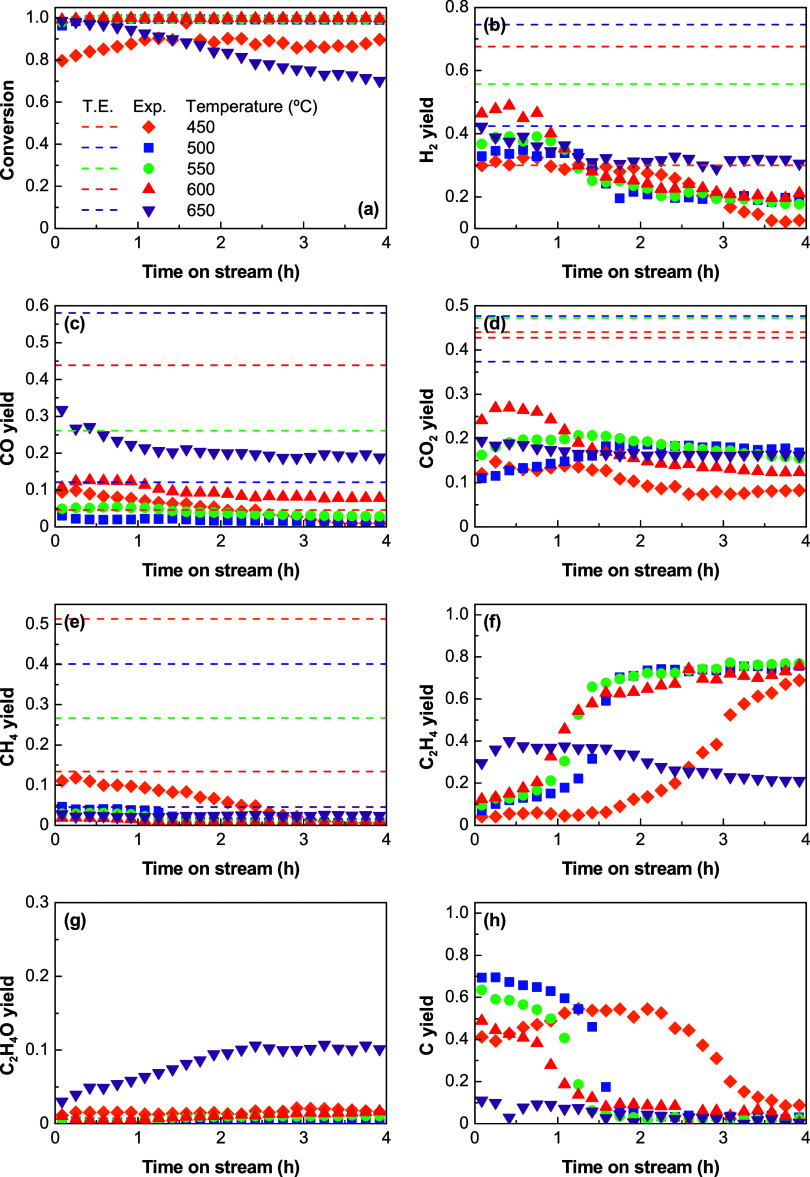
Effect of temperature at low space time on the evolution of (a) ethanol conversion and yields of (b) H_2_, (c) CO, (d) CO_2_, (e) CH_4_, (f) C_2_H_4_, (g) C_2_H_4_O, and (h) carbon/coke with time on stream over the Ni/Al_2_O_3_ catalyst derived from the NiAl_2_O_4_ spinel. Dashed lines correspond to the thermodynamic equilibrium predictions. Reaction conditions: S/E ratio, 3; space time, 0.025 h.

**Figure 3:**
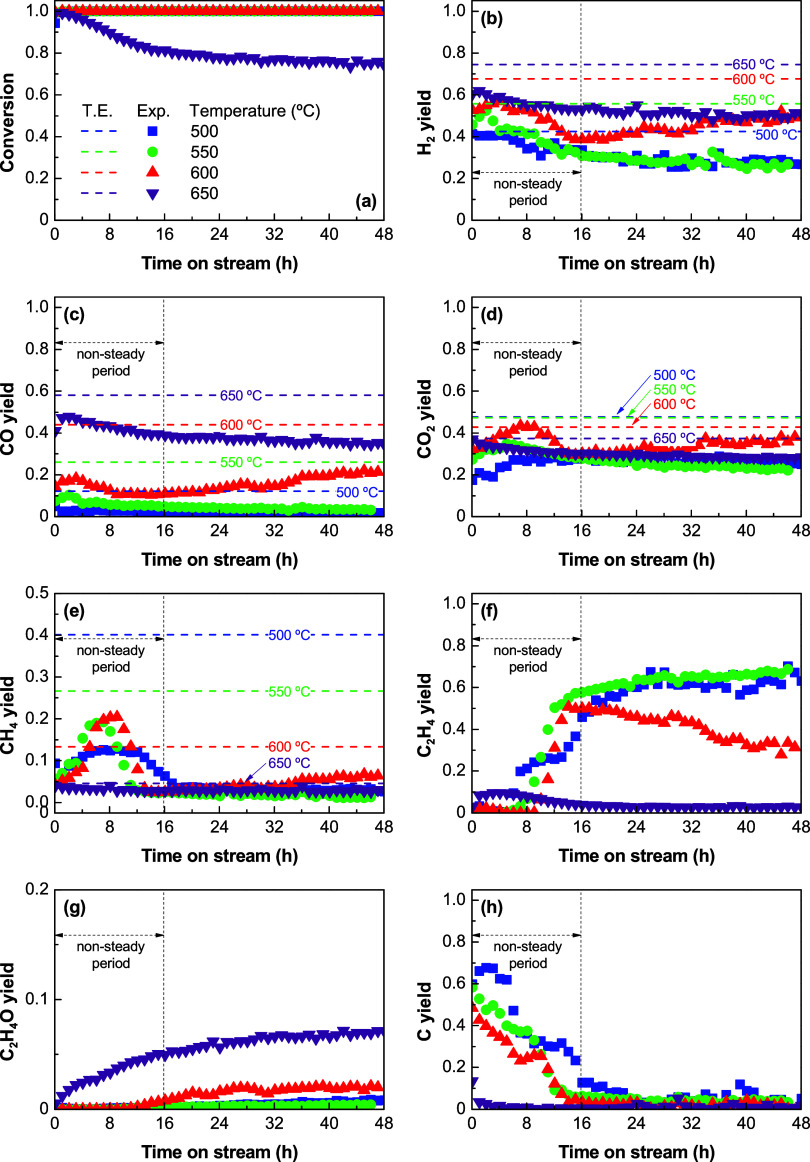
Effect of temperature at high space time on the evolution of (a) ethanol conversion and yields of (b) H_2_, (c) CO, (d) CO_2_, (e) CH_4_, (f) C_2_H_4_, (g) C_2_H_4_O, and (h) carbon/coke with time on stream over the Ni/Al_2_O_3_ catalyst derived from the NiAl_2_O_4_ spinel. Dashed lines correspond to the thermodynamic equilibrium predictions. Reaction conditions: S/E ratio, 3; space time, 0.1 h.

The effect of temperature on the evolution of the ethanol conversion and product yields with time was studied at two different space time values and for a constant S/E ratio of 3. Figure 2 shows the results for a low space time (0.025 h) at which the reaction is in a kinetic regime (so that ethanol conversion and the product yields have not reached their maxima). At these conditions, the effect of the catalyst deactivation by coke on the product distribution is noticeable, and this allows to observe the evolution of the product yields over a short time on stream. Figure 3 shows the results for a high space time (0.1 h), which is closer to an equilibrium regime (as observed in Section 3.1) and thus requires a longer time on stream (48 h in this run) to observe a comprehensive evolution of the product yields with time on stream. The horizontal dashed lines in the graphs of Figures 2 and 3 correspond to the yields predicted by the thermodynamic study. Due to various similarities for both conditions, the results are discussed simultaneously with the main focus on the phenomenon of the (partial) catalyst deactivation. The effect of temperature on the product distribution at zero time on stream was previously discussed (comments on Figures 1 and S2), and some inferences regarding the thermodynamics and thermally favored reactions were already mentioned.

Between 450 and 600 °C, the ethanol conversion (Figures 2a and 3a) is complete at zero time, even under low space time conditions, and remains complete over time on stream. However, at 650 °C, the conversion decreases over time on stream, evidencing a partial catalyst deactivation to convert ethanol at this temperature. The decrease in ethanol conversion is accompanied by the decrease in the C_2_H_4_ yield (Figures 2f and 3f) and increase in the C_2_H_4_O yield (Figures 2g and 3g). This may indicate a rapid deactivation of the Al_2_O_3_ acid sites to catalyze the ethanol dehydration (eq 4), and it also gives evidence of a change in the reaction routes at 650 °C, with C_2_H_4_O being an important intermediate at this temperature because its formation by ethanol dehydrogenation (eq 5) is thermally promoted at 650 °C (Figure S1). The selective deactivation of the catalyst acid sites for the dehydration of ethanol promotes its conversion to acetaldehyde by dehydrogenation both thermally and over the Ni sites, which explains the progressive increase in the yield of acetaldehyde over time at 650 °C. However, the slow but progressive decrease in the yields of H_2_, CO, and CO_2_ with time on stream suggests that, besides Al_2_O_3_ acid sites, the Ni active sites (where SR and WGS reactions occur) also become partially deactivated at 650 °C.

In spite of the sustained ethanol conversion over time on stream in the 450–600 °C range (Figures 2a and 3a), a partial catalyst deactivation is also evident in this temperature range according to the evolution of the product distribution with time on stream. Thus, catalyst deactivation causes a progressive decrease in the extent of the SR reactions (Table 1), which decreases the H_2_ yield with time on stream (Figures 2b and 3b). However, the trends in the evolution of the different products with time on stream are different for a given temperature value, which evidence a selective deactivation of the reactions summarized in Table 1. There is a relationship between the evolution of the yields of the main products (H_2_ (Figures 2b and 3b), C_2_H_4_ (Figures 2f and 3f), and carbon (Figures 2h and 3h)) with time on stream, showing a sharp change at a given time on stream. Accordingly, the H_2_ and carbon yields decrease while the C_2_H_4_ yield increases, similar to that described in a previous work.^28^ Thus, at the beginning of the reaction, there are high yields of H_2_ (Figures 2b and 3b) and carbon (Figures 2h and 3h) because the catalyst is highly active for ethanol dehydration (eq 4) and subsequent C_2_H_4_ decomposition (eq 8). It is then partially deactivated for C_2_H_4_ decomposition, as evidenced by the simultaneous decrease in the carbon and H_2_ yield and increase in the C_2_H_4_ yield. For a low space time (kinetic regime), the decrease in the H_2_ and carbon yields (Figures 2b and 2h, respectively) and increase in the C_2_H_4_ yield (Figure 2f) are brought forward over time with increasing temperature values, which evidence that the increase in the temperature speeds up this partial catalyst deactivation. Afterward, the yields of H_2_, C_2_H_4_, CO_2_, and CO reach a pseudostable state below 600 °C, indicating that the catalyst keeps a high activity for the ethanol dehydration (eq 4), WGS (eq 3), and SR reactions (eqs 1, 2, and 8–10). Interestingly, at 600 °C, once the catalyst is deactivated for the C_2_H_4_ decomposition reaction (from 16 h on stream), the yields of H_2_, CO, and CO_2_ slowly increase (Figure 3b–d), whereas that of C_2_H_4_ decrease (Figure 3f). This result suggests that the acid sites also deactivate slowly at this temperature, thus promoting the SR of ethanol, followed by the WGS reaction over the Ni sites.

**Figure 4:**
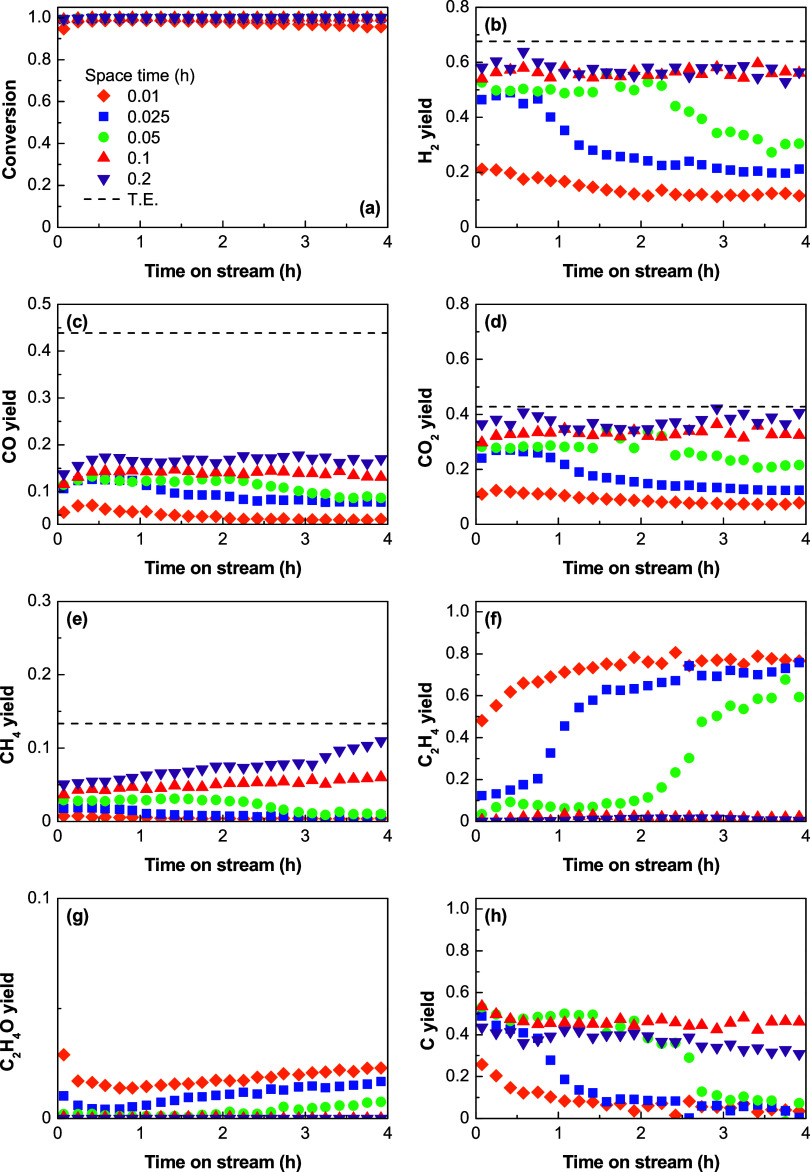
Effect of space time at 600 °C on the evolution of the (a) conversion and yields of (b) H_2_, (c) CO, (d) CO_2_, (e) CH_4_, (f) C_2_H_4_, (g) C_2_H_4_O, and (h) carbon with time on stream over the Ni/Al_2_O_3_ catalyst derived from the NiAl_2_O_4_ spinel. Reaction conditions: S/E ratio, 3.

The integration of the curves of the carbon yield shown in Figures 2h and 3h provides an estimation of the total amount of carbon formed at the end of each reaction (results in Figure S3a). As observed, the total amount of carbon decreases continuously with the increase in temperature at both space time values. It is noticeable that although the carbon yield (Figures 2h and 3h) and, consequently, the total carbon amount (Figure S3a), reach the lowest value at 650 °C compared to the reactions at lower temperature values, it has a high impact on the catalyst performance as it leads to a decrease in ethanol conversion. This can be explained by the different nature of the carbon formed and eventually deposited over the catalyst (coke) at this temperature, as it is well-known that the coke nature has a strong effect on the catalyst deactivation.^16,29,42^ Thus, unlike the carbon formed in the 450–600 °C range by the aforementioned C_2_H_4_ decomposition, at 650 °C, the coke origin is presumably the decomposition/cracking of C_2_H_4_O (eq 15). This agrees with the literature reports about the study of coke deposition on Ni catalysts used in the ESR, in which C_2_H_4_O has been defined as a coke precursor.^29^ Likewise, the coke nature has been associated with its origin: filamentous or nanostructured carbon (commonly carbon nanotubes) formed from the Boudouard reaction or the C_2_H_4_ and CH_4_ decompositions, and amorphous/turbostratic formed from C_2_H_4_O that blocks the Ni and Al_2_O_3_ sites.^16,29^

The formation of nanostructured carbon could be explained by a three-step mechanism, similar to the vapor–solid–solid (VSS) mechanism proposed for the growth mechanism of single-walled carbon nanotubes (SWCNTs).^47,48^ This involves the dissociation of the gaseous carbon precursor (C_2_H_4_, CH_4_, or CO) on the surface of the catalytic particle, the surface diffusion of carbon atoms on the solid particle, and the precipitation of carbon from the metal particles when the carbon solubility limit in the metal is reached. On the other hand, the formation of amorphous/turbostratic carbon from C_2_H_4_O is in agreement with the observation of Kontchouo et al.,^49^ who recently reported that acetaldehyde is strongly adsorbed on the surface of a Ni catalyst and leads to rapid polymerization even at very low temperatures, forming a polymeric coke of highly aliphatic nature.

These results provide relevant information for the scale-up of the ESR process. As observed in Figures 2 and 3 after 2 or 16 h on stream, respectively, the catalyst keeps a high stability reaching a pseudosteady state with constant yields of H_2_ and byproducts. Exceptionally, the yields slightly vary in this pseudosteady state at 600 °C, which may be a consequence of the incipient selective catalyst deactivation for the ethanol dehydration that promotes the SR and WGS reactions (explaining the progressive increase in the H_2_ and CO yields).

The peculiar trend of CH_4_ yield at high space time (Figure 3e) is noted, which is low at the beginning of the reactions and increases with time on stream, reaching a maximum value, and then decreases to very low values (almost negligible) when the C_2_H_4_ decomposition reaction is over. This is evidenced by the constant (below 600 °C) or decreasing (at 600 °C) values of the C_2_H_4_ yield in Figure 3f. This trend has also been reported by Sanchez-Sanchez et al.,^50^ and the maximum in the evolution of the CH_4_ yield in the 500–600 °C range reveals that there are various routes forming or consuming CH_4_ during the catalyst deactivation period. Presumably, CH_4_ may be formed initially from the decomposition of ethanol (eq 6) and C_2_H_4_O (eq 7) or from the methanation reaction (reverse of eq 10) though these reactions proceed to a limited extent according to the data in Figure 2. Moreover, CH_4_ may be formed from C_2_H_4_, according to our previous experimental observation, when we evaluated the ethylene decomposition on the same catalyst at 500 and 600 °C,^28^ and where CH_4_ was also formed together with carbon and H_2_. Malaika and Kozłowski^51^ reported similar observations and proposed the partial decomposition of C_2_H_4_ (eq 19) as a plausible route for the CH_4_ formation. Likewise, we propose an alternative for the formation of CH_4_ by C_2_H_4_ hydrogenolysis (eq 20) that probably may not be direct, but it may proceed through the C_2_H_4_ hydrogenation (eq 21), followed by the C_2_H_6_ hydrogenolysis (eq 22)^52^
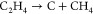
19
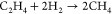
20
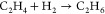
21
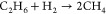
22According to the data in Figure 3, the phenomenon of the catalyst deactivation may be related to the selective decrease in the extent of individual reactions in Table 1. At zero time on stream with enough active sites (near-equilibrium regime), the catalyst has the sufficient amount of active sites for the complete C_2_H_4_ decomposition (eq 11) but the extent of this reaction decreases rapidly with time on stream because the active sites are progressively occupied by carbon.^28^ Then, when the catalyst deactivates for the C_2_H_4_ decomposition, other reaction routes for the conversion of C_2_H_4_ are promoted, presumably the hydrogenolysis (eq 20) and the partial decomposition (eq 19), which explains the decrease in the carbon yield (Figure 3h) and the increase in the CH_4_ yield (Figure 3e). As the active sites become completely saturated with carbon, the CH_4_ and carbon yields simultaneously undergo a sharp decay, leaving a maximum in the CH_4_ yield, which indicates the apparent termination of the C_2_H_4_ decomposition reactions (eqs 11 and 19), as well as the deactivation of the C_2_H_4_ hydrogenolysis reaction (eq 20).

At the same time, carbon may be gasified by H_2_O (eq 16) and the CO formed is converted into CO_2_ by the WGS reaction (eq 3), which explains the increasing evolution of the CO_2_ yield with time on stream (Figure 3d), particularly at 550 and 600 °C (since gasification would be more favored at these high-temperature values). The subsequent decreasing trend in the CO_2_ yield may indicate that the carbon gasification rate reaches a maximum in the first period of the reaction run, coinciding with carbon formation by the decomposition of C_2_H_4_ (eq 11). Afterward, the carbon gasification is presumably slowed down and the carbon remaining on the catalyst is more stable (resistant to be gasified).

Additionally, it should be considered the particular dynamics of the saturation of Ni sites with carbon filaments/nanotubes, causing a catalyst restructuration, in which Ni sites are detached from the Al_2_O_3_ support and exposed on the tips of the carbon filaments.^28,38,53^ As a result of this relocation of the Ni sites, although the catalyst is deactivated for the C_2_H_4_ conversion through decomposition and hydrogenolysis reactions (eqs 11, 19, and 20), it keeps a high activity for other reactions of Table 1, forming H_2_, CO, CO_2_, and CH_4_ (on Ni sites) and C_2_H_4_ (on acid sites), as previously commented.

#### 3.2.2 Space Time

Figure 4 shows the effect of space time on the evolution of the ethanol conversion and product yields with time on stream at 600 °C and at an S/E ratio of 3. These results and those obtained at 500 °C (Figure S4) are discussed concurrently due to their similarities. It is evident that the conversion is complete at both temperatures (Figures 4a and S4a) for a space time above 0.025 h, and this conversion is constant over the whole reaction run (4 h on stream). Based on kinetics grounds, the increase in the space time implies an increase in the Ni and Al_2_O_3_ sites, which increases the initial yields of H_2_, CO, CO_2_, and CH_4_ (Figures 4b–e and S4b–e, respectively) as the extent of the their formation reactions (Table 1) is promoted. However, as discussed in Section 3.1, the experimental yields at zero time on stream generally do not reach the predicted values for the thermodynamic equilibrium (represented by the dashed lines in each plot). It should be noted that the carbon yield at zero time on stream (Figure 4h) reaches a maximum value (for a space of time between 0.05 and 0.1 h). This suggests that the increase in the amount of active sites favors the extent of SR, dehydrogenation to C_2_H_4_O, decomposition, and WGS reactions over the dehydration to C_2_H_4_ and subsequent decomposition to carbon and H_2_.

The space time significantly affects the evolution over time on the stream of the yields of gaseous products and carbon. The increase in the C_2_H_4_ yield (Figures 4f and S4f) occurs later as the space time increases and is not observed at a space time of 0.1 h and above in the period shown in these Figure 4h. The comparison of these results with the evolution of the carbon yield (Figures 4h and S4h) confirms the hypothesis of the carbon formation mainly from C_2_H_4_ in the 500–600 °C range since the decrease in the carbon yield coincides with the increase of its precursor (C_2_H_4_) in the gas phase. Accordingly, the C_2_H_4_ yield does not increase in the studied period for high space times (≥0.1 h) due to the high availability of active sites for its conversion by total or partial decomposition (eqs 11 and 19, respectively) or hydrogenolysis (eq 20). Likewise, the carbon yield at these high space time values shows just a slight decrease that may be related to the increase in the CH_4_ yield (Figures 4e and S4e), which suggests the selective deactivation of the catalyst for the complete decomposition of C_2_H_4_ (eq 11), which favors its partial decomposition (eq 19) or hydrogenolysis (eq 20).

Based on these results, it can be assumed that the effect of catalyst deactivation on the product distribution can be delayed by increasing the space time. This behavior is characteristic of a deactivation mechanism that takes place in parallel with the SR reactions (desired reactions) since the amorphous carbon (coke) that deactivates the catalyst is formed by degradation of intermediates (C_2_H_4_ (eq 14) up to 600 °C and above this temperature also C_2_H_4_O (eq 15)) that are formed rapidly and directly from ethanol. Moreover, when the space time is increased, there are more active sites for the formation of nanostructured carbon by C_2_H_4_ decomposition (on remaining Ni sites strongly interacting with the Al_2_O_3_ support), and therefore, the extent of this reaction would be prolonged over time. These results are consistent with the role of coke in causing catalyst deactivation for all of the reactions summarized in Table 1, including its formation reactions from C_2_H_4_ (eq 14) and C_2_H_4_O (eq 15) catalyzed by the acid sites of the Al_2_O_3_ support.

#### 3.2.3 Steam/Ethanol (S/E) Ratio

**Figure 5:**
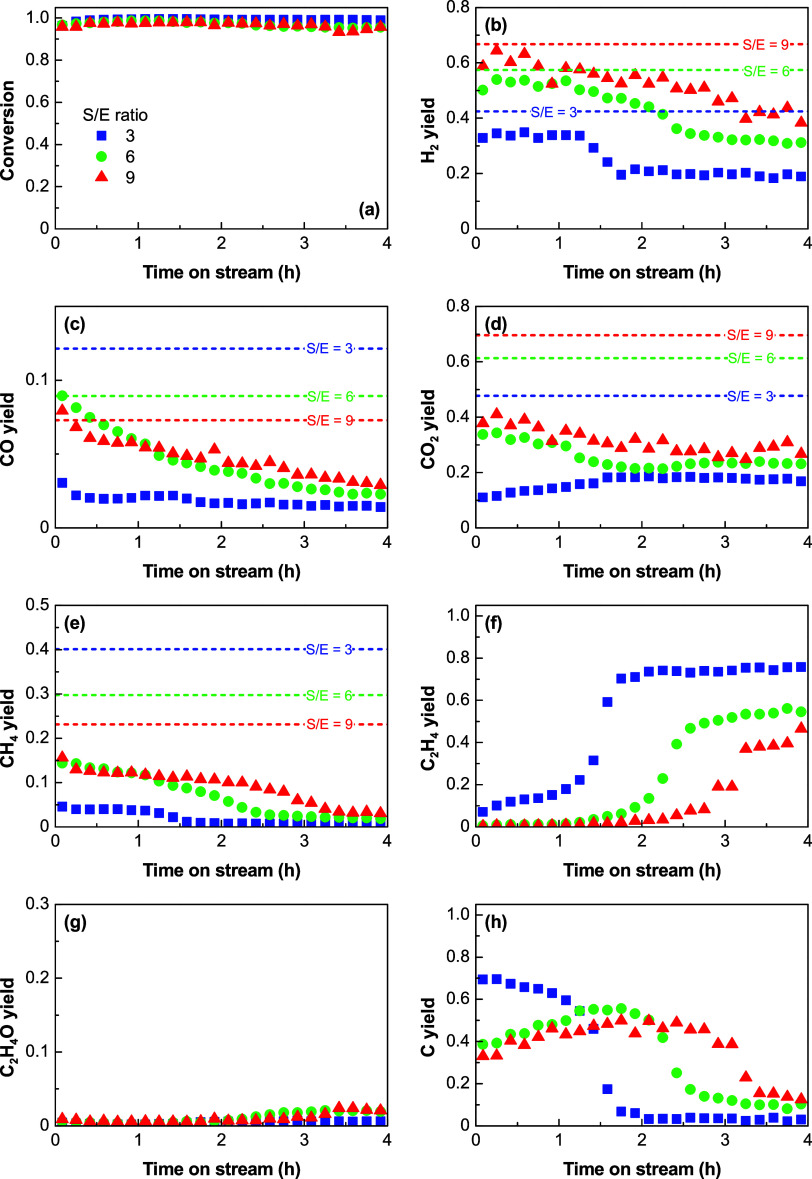
Effect of S/E ratio on the evolution of the (a) conversion and yields of (b) H_2_, (c) CO, (d) CO_2_, (e) CH_4_, (f) C_2_H_4_, (g) C_2_H_4_O, and (h) carbon with time on stream over the Ni/Al_2_O_3_ catalyst derived from the NiAl_2_O_4_ spinel. Dashed lines correspond to the thermodynamic equilibrium. Reaction conditions: 500 °C; space time: 0.025 h.

The effect of the S/E ratio on the catalyst stability has been studied with a low space time of 0.025 h (kinetic regime), and the corresponding evolution of the ethanol conversion and product yields with time on stream is shown in Figures 5 (500 °C) and S5 (600 °C). The use of a low space time allows observing more pronounced differences in the catalyst stability than with a high space time. The increase in the S/E ratio causes an increase in the yields at zero time of H_2_ (Figures 5b and S5b) and CO_2_ (Figures 5d and S5d) and also a decrease in the carbon yield (Figures 5h and S5h), which is particularly evident at 600 °C (Figure S5h) due to the additional effect of the promoted carbon gasification.

Regarding the catalyst stability, the results at both temperatures show that ethanol is fully converted during 4 h on stream for all of the S/E ratio values tested. Additionally, the increase in the S/E ratio results in a more stable evolution of the product yields with time on stream, especially H_2_ (Figures 5b and S5b), C_2_H_4_ (Figures 5f and S5f), and carbon (Figures 5h and S5h). The carbon yield decreases with time on stream concurring with the increase in the C_2_H_4_ yield, which confirms the hypothesis aforementioned about the origin of carbon formation by the C_2_H_4_ decomposition. Additionally, the yield of CH_4_ (Figures 5e and S5e) also shows an abrupt decrease concurrent with the increase in C_2_H_4_ yield, which evidence that it is mainly formed from C_2_H_4_ (the main intermediate compound at 500 °C) by partial decomposition (eq 19) and/or hydrogenolysis (eq 20), as commented in Sections 3.2.1 and 3.2.2.

The results in Figure 5b,f,h evidence that the increase in the H_2_O concentration in the reaction medium attenuates the catalyst deactivation for the C_2_H_4_ decomposition reaction (eq 11), which is the main carbon forming reaction. As a result, the total duration of carbon formation by this route is prolonged, which leads to a higher amount of carbon formed at the end of the reaction run, as shown in Figure S3b. This result may be explained by assuming that carbon is formed on Ni sites with strong interaction with Al_2_O_3_ and the growth of the carbon filaments detaches the Ni sites from the support, thus decreasing the Ni–Al_2_O_3_ interaction and causing Ni sites to be exhausted (no longer active) to form carbon filaments. For the same number of Ni sites initially available, the faster the rate of carbon formation, the faster the Ni sites will be exhausted. The increase in the water concentration by increasing the S/E ratio slows down and attenuates the carbon formation initially, which delays the exhaustion of Ni sites for the carbon formation compared to a low S/E ratio, and this prolongs the carbon formation over time. Consequently, the rapid carbon formation with an S/E ratio of 3 makes the Ni sites to be exhausted in about 1.4 h on stream, whereas the slower carbon formation rate with an S/E ratio of 9 makes the Ni sites to be exhausted in about 3 h on stream.

It should be noted that the high carbon yields are also attributable to the limited extent of the carbon gasification at 500 °C. This effect of the increase in the H_2_O concentration is different from ESR reactions on other catalysts, in which ethanol dehydration and subsequent C_2_H_4_ decomposition are not relevant reaction routes in the carbon formation.^17,30,54^ In these cases, the total amount of carbon formed decreases with the increase in the S/E ratio. Conversely, the effect of increasing the H_2_O concentration on promoting the overall carbon formation is not observed at 600 °C (Figure S5h), because the SR (of ethanol and all intermediates, including C_2_H_4_, CH_4_, and C_2_H_4_O) and carbon gasification reactions are more favored than the ethanol dehydration and subsequent C_2_H_4_ decomposition. Consequently, at 600 °C the total amount of carbon formed at the end of the reaction decreases with the increase in the S/E ratio (Figure S3b).

Curiously, the CH_4_ yield does not drop down to negligible values with a high H_2_O concentration at 600 °C (Figure S5e), and the values are even higher than the predicted values in thermodynamic equilibrium at this temperature. This suggests that CH_4_ continues to be formed over time on stream by methanation (reverse of eq 10) of the CO formed by carbon gasification (strongly favored with the increase in S/E ratio at 600 °C), which would explain the higher CH_4_ yields with increasing H_2_O concentration, although the SR reactions are also favored.

**Figure 6:**
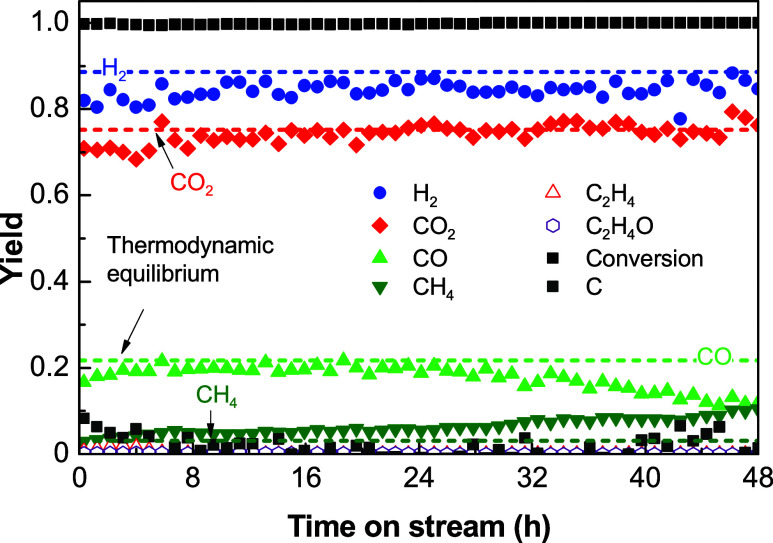
Evolution of the ethanol conversion and product yields with time on stream over the Ni/Al_2_O_3_ catalyst derived from the NiAl_2_O_4_ spinel. Reaction conditions: 600 °C; space time, 0.1 h; S/E ratio, 9.

It is noteworthy that the high and stable H_2_ yield (around 70%) was obtained at 600 °C with an S/E ratio of 9 and a low space time of 0.025 h (Figure S5b). According to the effect shown in Section 3.2.2, the increase in the space time would contribute to obtain a higher H_2_ yield and even with a more stable behavior, as shown in Figure 6, corresponding to a long duration reaction run at 600 °C with a space time of 0.1 h and with an S/E ratio of 9. As observed, a stable yield of H_2_ around 85% is obtained over 48 h on stream, with a low formation of carbon in the whole reaction run, which allows attaining yields of gaseous products close to the thermodynamic equilibrium (dashed lines). Comparing the performance of the catalyst used in this work with others based on Ni, Montero et al.^17^ obtained a H_2_ yield of 82% at 600 °C and 80% at 650 °C with an S/E ratio of 6 and a space time of 0.35 h on a Ni/La_2_O_3_-αAl_2_O_3_ catalyst. At these conditions, the catalyst was stable for 200 h, and the yields of CO_2_, CO, and CH_4_ were, respectively, 64, 22, and 13% at 600 °C and 60, 32, and 6% at 650 °C. Vicente et al.^30^ reported a stable behavior of a Ni/SiO_2_ catalyst for 20 h at 700 °C, an S/E ratio of 6, and a space time of 0.18 h, obtaining a H_2_ yield of 80% and yields of CO_2_, CO, and CH_4_ of 53, 40, and 10%, respectively.

### 3.3 Global Vision of the Effect of Reaction Conditions on the Reaction Routes

**Figure 7:**
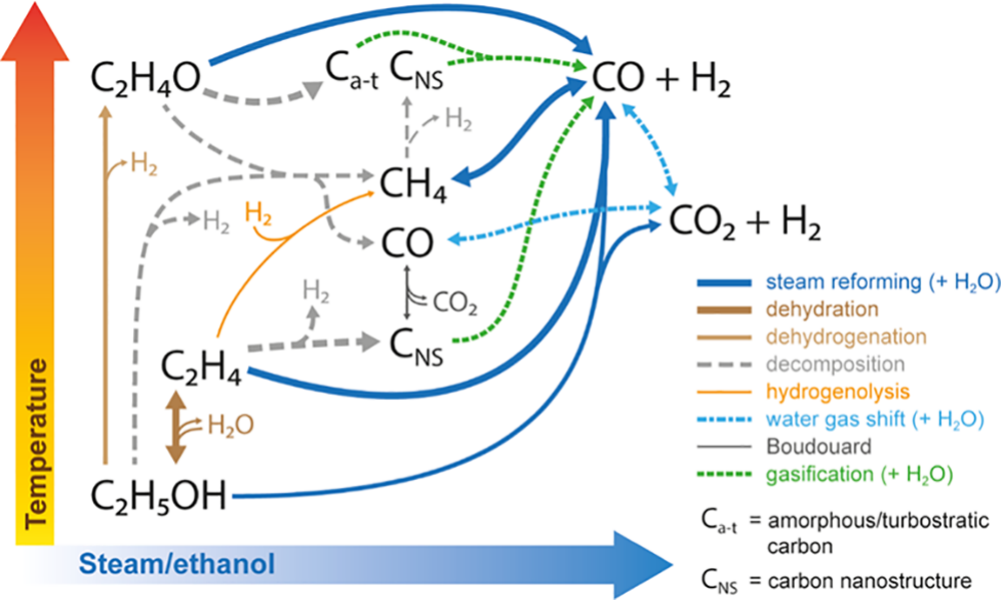
Effect of reaction conditions (temperature and S/E ratio) on the extent of the reactions in the ESR on a Ni/Al_2_O_3_ catalyst derived from the NiAl_2_O_4_ spinel.

Figure 7 shows a general scheme for the main reactions taking place in the ESR and summarizes the analyses in the previous sections about the effect of reaction conditions (temperature and S/E ratio) on the extent of each reaction for the formation of gaseous and solid products, taking into consideration the known acidic properties of the catalyst.^28^

With a low H_2_O concentration in the feed, ethanol is mainly dehydrated to C_2_H_4_ at 450–600 °C and both dehydrated and dehydrogenated to C_2_H_4_O at 650 °C. However, ethanol decomposition to CH_4_, CO, and H_2_ and ethanol SR to CO/CO_2_ and H_2_ also occur to some extent, with the extent of the latter reaction significantly increasing as the S/E ratio is increased. C_2_H_4_ undergoes mainly decomposition to nanostructured carbon (C_NS_) and H_2_, which is a relevant route at low-temperature and S/E ratio values. Partial decomposition and hydrogenolysis of C_2_H_4_ (via hydrogenation to C_2_H_6_) may occur, thus forming CH_4_. Concurrently, C_2_H_4_, C_2_H_4_O, and CH_4_ undergo SR to CO and H_2_, and these reactions being favored at high-temperature and S/E ratio values. Likewise, the WGS reaction is favored at low/mild temperature values and high S/E ratio values, which is presumably a fast reaction. The C_NS_ may be also formed at high temperatures by CH_4_ decomposition and at low temperatures by the Boudouard (CO disproportionation) reaction, although the reverse of the latter reaction would gasify this carbon at high-temperature values. Moreover, at high temperatures, a different type of carbon (amorphous/turbostratic, C_a–t_) may be also formed from C_2_H_4_O. The gasification of both C_NS_ and C_a–t_ with H_2_O to CO and H_2_ is a relevant reaction, especially at high-temperature and S/E ratio values, which has a significant impact on the carbon yield and consequently on the yield and distribution of carbonaceous gas products.

The rate of catalyst deactivation depends on the content and nature of the solid carbon formed, which is also dependent on the temperature and S/E ratio. The carbon nature has been defined in this work as amorphous/turbostratic carbon (C_a–t_) and nanostructured carbon (C_NS_) based on the observations made with scanning electron microscopy (SEM) of spent catalyst samples (Figures S6 and S7). In brief, the SEM images clearly show the abundant presence of nanostructured carbon in the form of filaments at 500 °C (Figures S6b and S7b) and the poor presence of carbon filaments at 650 °C with some amorphous mass of solid carbon (Figures S6b and S7b). This mass of carbon may be a turbostratic carbon based on the high temperature (650 °C) at which it was formed. Thus, in regards to the catalyst deactivation, it is known that the blockage of Ni and Al_2_O_3_ acid sites is mostly due to the deposition of amorphous/turbostratic carbon, whereas it barely occurs when nanostructured carbon (filaments or nanotubes) is formed.^17,18^

**Figure 8:**
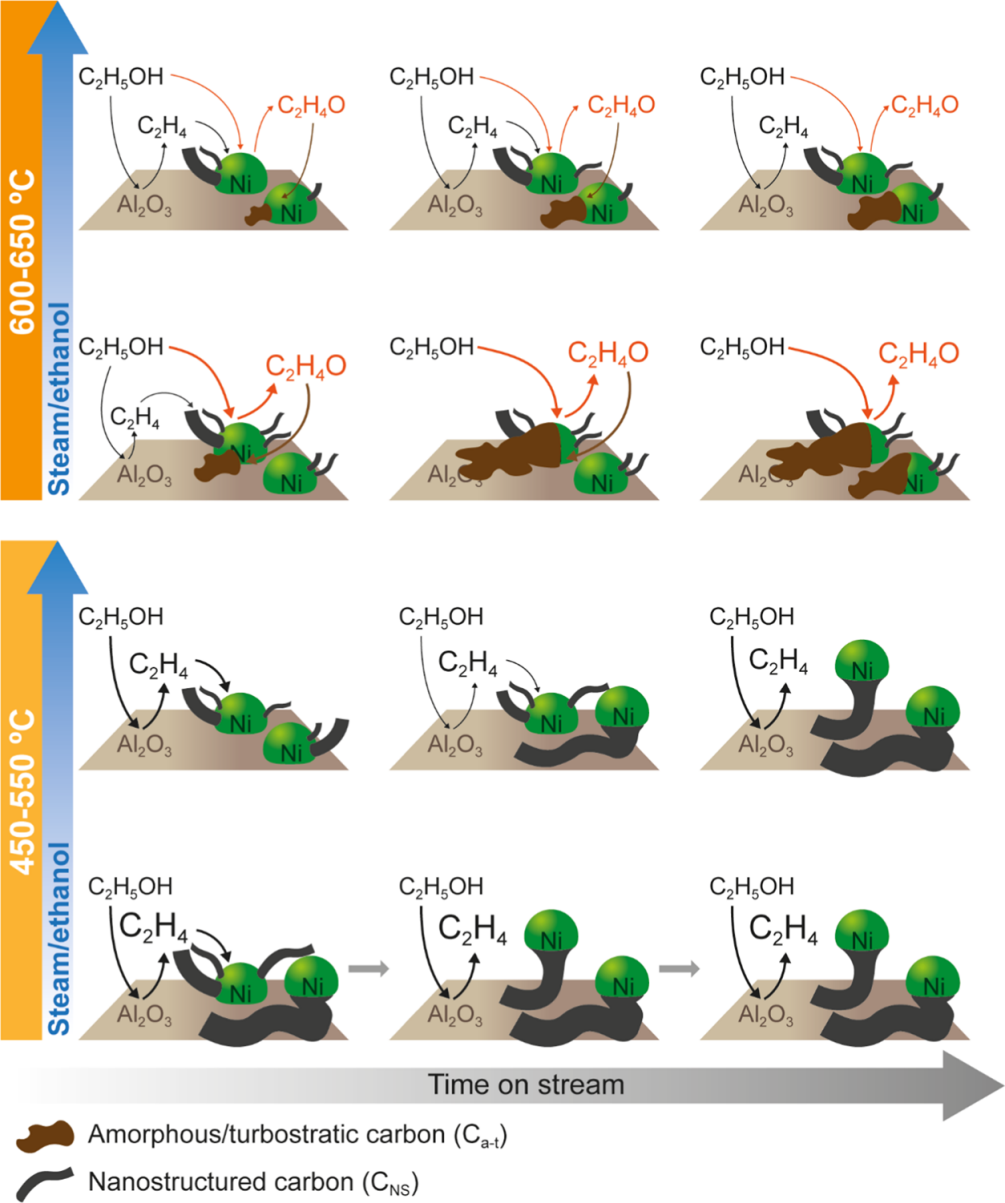
Effect of the reaction conditions (temperature and S/E ratio) on the evolution of carbon formation with time on stream.

Figure 8 shows a scheme for the effect of the reaction conditions on the formation dynamics of both carbon types. The formation of C_NS_ carbon is predominant at low-temperature and S/E ratio values, at which ethanol is mainly dehydrated to C_2_H_4_ on Al_2_O_3_ acid sites and, subsequently, C_2_H_4_ is decomposed to these carbon nanostructures on the Ni sites (see the lower sequence in Figure 8). Over time, the C_NS_ carbon continues to grow and cause the Ni crystals to be detached from the support. In this stage, the C_2_H_4_ decomposition may be partial, generating more CH_4_ in the gaseous products as revealed by the product analyses at 500–600 °C (Figures 2e and 3e). When the carbon nanostructures have grown on all of the Ni sites (possibly reaching a saturation state at which no more carbon filaments can be formed), C_2_H_4_ is no longer converted. This suggests that C_2_H_4_ formed on Al_2_O_3_ sites would not be effectively adsorbed on Ni sites to continue reacting because of the separation of Ni sites from the support (since a close interaction between Ni and Al_2_O_3_ is required to convert C_2_H_4_ by this route). Thus, when the catalyst is in this state, ethanol continues to be converted on Al_2_O_3_ sites generating C_2_H_4_, and the catalyst keeps the Ni sites active for the SR, dehydrogenation, decomposition, and WGS reactions (Table 1), yielding H_2_, CO, CO_2_, and CH_4_.

The role of the S/E ratio in attenuating the carbon formation reactions is relevant, and this role depends on the reaction temperature. Thus, up to 550 °C, at which C_2_H_4_ is the main intermediate in the reaction network and the extent of carbon gasification is limited, the increase in the S/E ratio has a dual role. On the one hand, it attenuates the C_2_H_4_ formation by dehydration because other H_2_O-consuming reactions in Table 1 are promoted and, consequently, the initial carbon formation by C_2_H_4_ decomposition (eq 11) is also slowed down (as evidenced in Figures 5h and S5h). On the other hand, it attenuates the deactivation of the C_2_H_4_ decomposition reaction, which prolongs the nanostructured carbon formation by this route over time (upper sequence in the 450–550 °C range in Figure 8), leading to a higher total amount of carbon formed at high time on stream values. Conversely, at high-temperature values (in the 600–650 °C range), C_2_H_4_O is a relevant intermediate, as its formation is favored by ethanol dehydrogenation (especially at 650 °C, as evidenced in Figure S1). At these conditions, the formation of amorphous/turbostratic carbon from oxygenates (ethanol or C_2_H_4_O) preferentially deposited on Al_2_O_3_ sites hinders the ethanol adsorption on these sites and therefore slows down the C_2_H_4_ formation. However, the increase in the steam/ethanol ratio at this high temperature favors the gasification of the coke deposited on Ni sites and allows them to remain active for other reactions, yielding H_2_, CO, CO_2_, and CH_4_.

## 4 Conclusions

The ethanol steam reforming on a Ni/Al_2_O_3_ catalyst (derived from NiAl_2_O_4_ spinel) takes place through a complex reaction network, in which the extent of each reaction is determined by the catalyst deactivation and depends on the reaction conditions (temperature, space time, and S/E ratio). The selective deactivation of the Ni and Al_2_O_3_ acid sites for the different reactions, including the formation of nanostructured and amorphous/turbostratic carbon, explains the evolution of the product distribution with time on stream.

The ethanol dehydration to C_2_H_4_ (on the acidic Al_2_O_3_ sites) followed by C_2_H_4_ decomposition (on the Ni sites with strong interaction with the Al_2_O_3_ support) is the most relevant reaction pathway up to 550 °C, and high nanostructured (filamentous) carbon yields (maximum of 63%) can be obtained together with reasonably high H_2_ yields (about 53–43%). In this temperature range, the catalyst deactivation selectively affects the C_2_H_4_ decomposition reaction, causing an abrupt decrease in the carbon formation and H_2_ yield after a certain time on stream, so that the catalyst reaches a pseudosteady state with a remaining activity that is almost constant afterward. Above 600 °C, there is an apparent change in the prevailing route in the H_2_ formation in the reaction network, with C_2_H_4_O formed by ethanol dehydrogenation being a relevant intermediate (mainly at 650 °C) in the formation of H_2_ and amorphous/turbostratic carbon (coke) deposited on both acidic and Ni sites. This coke rapidly affects the ethanol dehydration on acid sites, but the favored extent of its gasification on Ni sites at a high temperature and S/E ratio allows these sites to keep a significant remaining activity for the SR and WGS reactions.

The increase in the H_2_O concentration (by increasing the S/E ratio) speeds up all of the reactions that have H_2_O as a reactant, including the SR and WGS reactions, which partially suppresses the ethanol dehydration to C_2_H_4_ and its subsequent decomposition reactions. This limits the extent of the reactions forming carbon/coke, which slows down the deactivation rate of the catalyst. The combined increase in temperature and S/E ratio is key to promoting the carbon/coke gasification and to maximizing the catalyst stability. A high H_2_ yield (around 85%) with a low carbon yield (below 5%) is obtained at 600 °C, with a space time of 0.1 h and an S/E ratio of 9. These conditions are relevant to scale up the ESR process as a constant H_2_ yield is attained for 48 h on stream, whose period can be prolonged by increasing the space time.

The aforementioned results are interestingly useful to develop a kinetic model for the ESR reaction (targeted at the scale-up of the reactor), which should be able to quantify the effects studied in this work on the rate of each reaction in the global reaction network and of catalyst deactivation.

## Supporting Information Available

Results of the ethanol steam thermal reaction; additional results of the ESR reaction tests; SEM images of the carbon formed on the catalyst (PDF)
