# Supplementary material for: Global Vision of the Reaction and Deactivation Routes in the Ethanol Steam Reforming on a Catalyst Derived from a Ni–Al Spinel
Source: Energy Fuels. 2024 Apr 9;38(8):7033–48. doi: 10.1021/acs.energyfuels.4c00646 (PMC11033872; doi:10.1021/acs.energyfuels.4c00646)
Supplement: Supplementary file 1 — ef4c00646_si_001.pdf [file ef4c00646_si_001.pdf]

# SUPPORTING INFORMATION

**This document contains supplementary results for the article:**

## Global vision of the reaction and deactivation routes in the ethanol steam reforming on a catalyst derived from Ni-Al spinel

Sergio Iglesias-Vázquez, José Valecillos*, Aingeru Remiro, Beatriz Valle, Javier Bilbao, Ana G. Gayubo*

Department of Chemical Engineering, University of the Basque Country (UPV/EHU) P.O. Box 644, Bilbao, 48080 Spain

(*) Corresponding author(s): jose.valecillos@ehu.eus; anaguadalupe.gayubo@ehu.eus

## 1. Thermal reaction

Figure S1 shows the molar fractions of components as a function of the temperature for the thermal reaction of ethanol (without catalyst) with S/E molar ratio of 3. In absence of a catalyst, ethanol is barely converted at the temperature range of 500-600 ºC and its conversion begins to be noticeable (ethanol fraction decreases) above 600 ºC yielding $H_2$, CO, $CH_4$, acetaldehyde ($C_2H_4O$) and ethylene ($C_2H_4$). Since the $H_2O$ fraction only decreases slightly, we can conclude that the main reactions taking place are the ethanol dehydration and dehydrogenation, and acetaldehyde decomposition (Eq. 7), whereas SR reactions seem to be negligible at these conditions (as evidenced by the almost equal amounts of CO and $CH_4$). As expected, the steam reforming and water gas shift reactions barely proceed thermally and a catalyst is needed in order to accelerate the rate of these reactions.

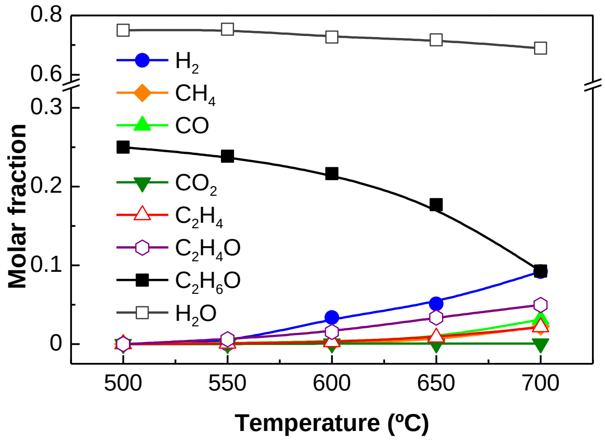


**Figure S1.** Effect of temperature on the product distribution ($N_2$ free molar fraction on a wet basis) for the thermal reaction of ethanol and steam. Conditions: S/E ratio of 3, ethanol partial pressure of 0.05 bar.

## 2. Catalytic reactions

Figure S2 shows the effect of the S/E ratio in the feed on the product distribution at equilibrium conditions (high space time) as a function of temperature. Figure S3 shows the amount/content of carbon formed in the catalytic bed after the reactions reported in Figures 2, 3 and 4 in the article, and S5 in this document. Figures S4 and S5 show the evolution of the products yields over time

for several ESR reactions using a $NiAl_2O_4$ derived catalyst under different reaction conditions. These results are further commented in the main text.

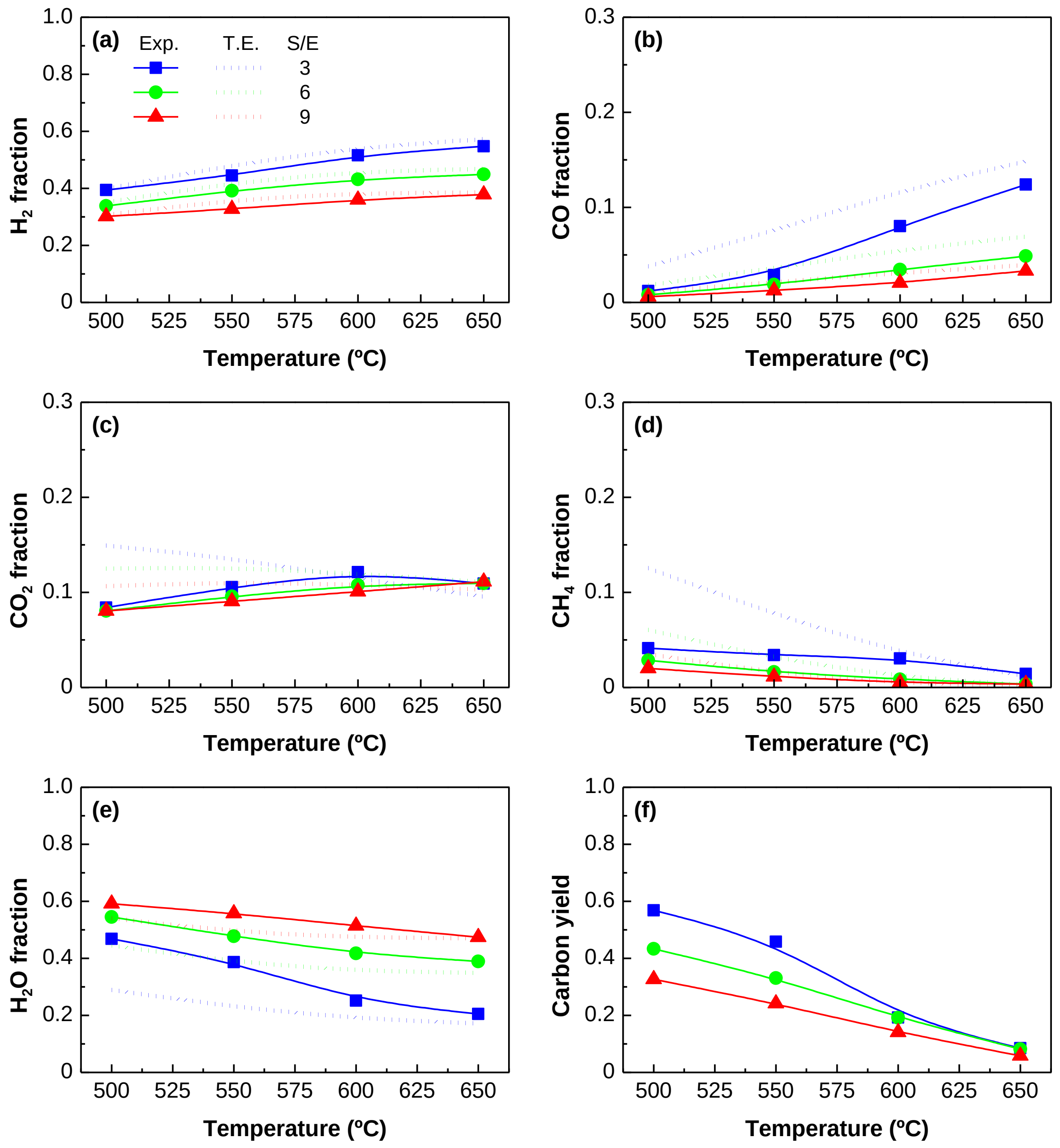


**Figure S2.** Effect of the S/E ratio in the feed on the product distribution ($N_2$ free molar fraction on a wet basis) as a function of temperature (solid lines) and comparison with the thermodynamic equilibrium predictions (dashed lines): (a) $H_2$, (b) CO, (c) $CO_2$, (d) $CH_4$, (e) $H_2O$. The yield of (f) carbon is calculated by C atom balance. Reaction conditions: space time, 1.3 h; ethanol partial pressure, 0.05 bar.

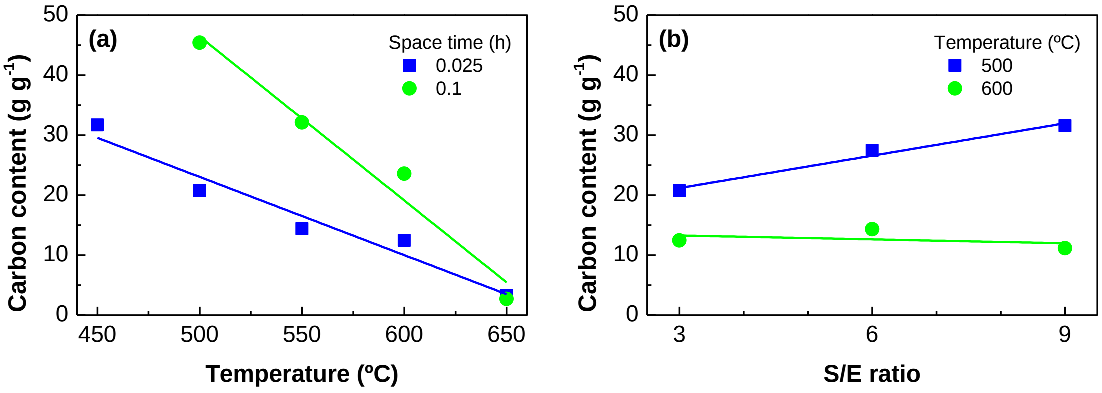


**Figure S3.** Effect of (a) temperature and (b) S/E ratio on the total amount of carbon/coke formed at the end of the ESR reaction. Values calculated by integration of the carbon yield evolutions. Reaction conditions: (a) S/E ratio of 3 and space time of 0.025 and 0.1 h; (b) space time of 0.025 h at 500 and 600 ºC.

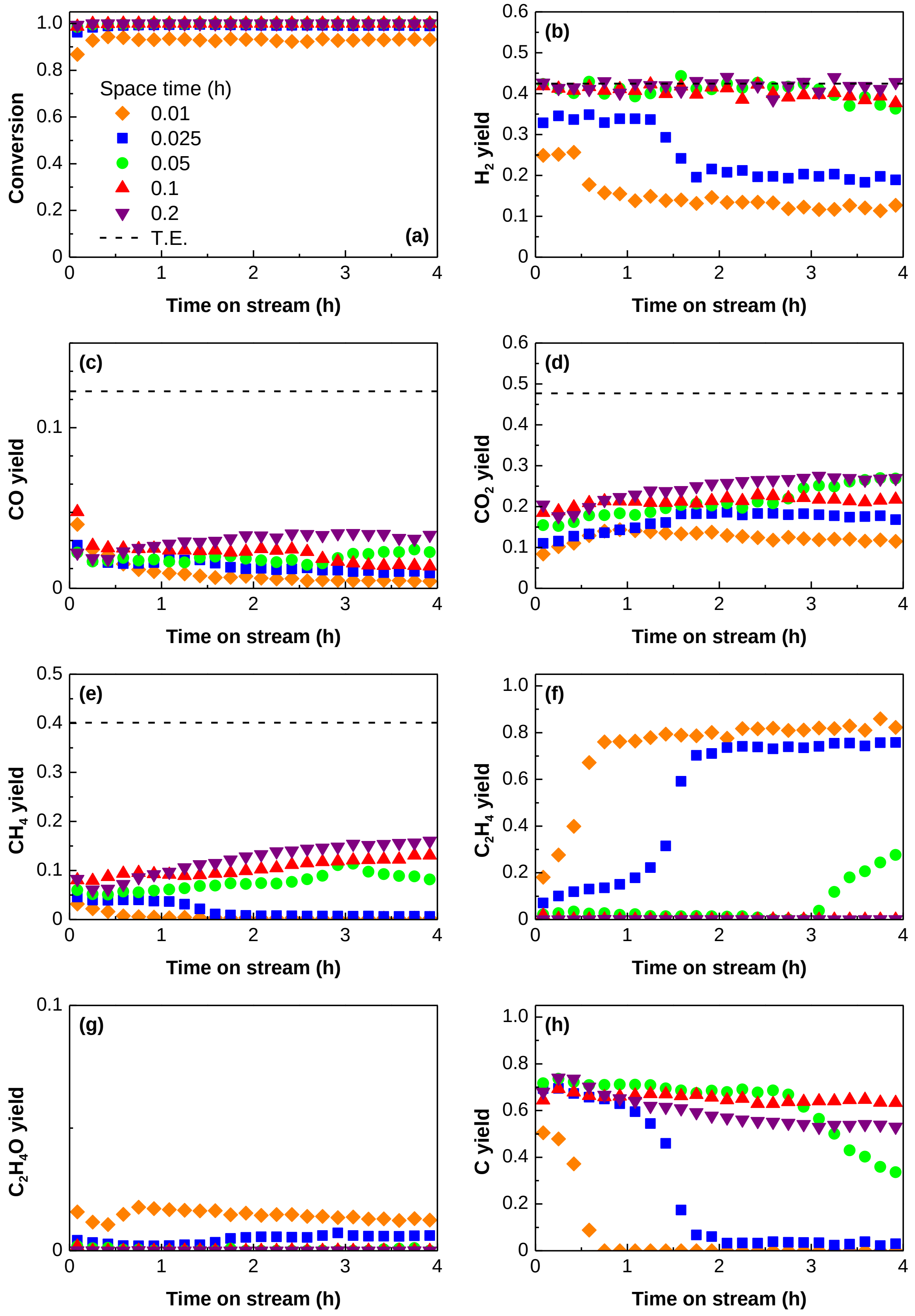


**Figure S4.** Effect of space time at 500 °C on the evolution of the (a) conversion and yields of (b) $H_2$, (c) CO, (d) $CO_2$, (e) $CH_4$, (f) $C_2H_4$, (g) $C_2H_4O$, and (h) carbon with time on stream. Reaction conditions: S/E ratio, 3; ethanol partial pressure, 0.05 bar.

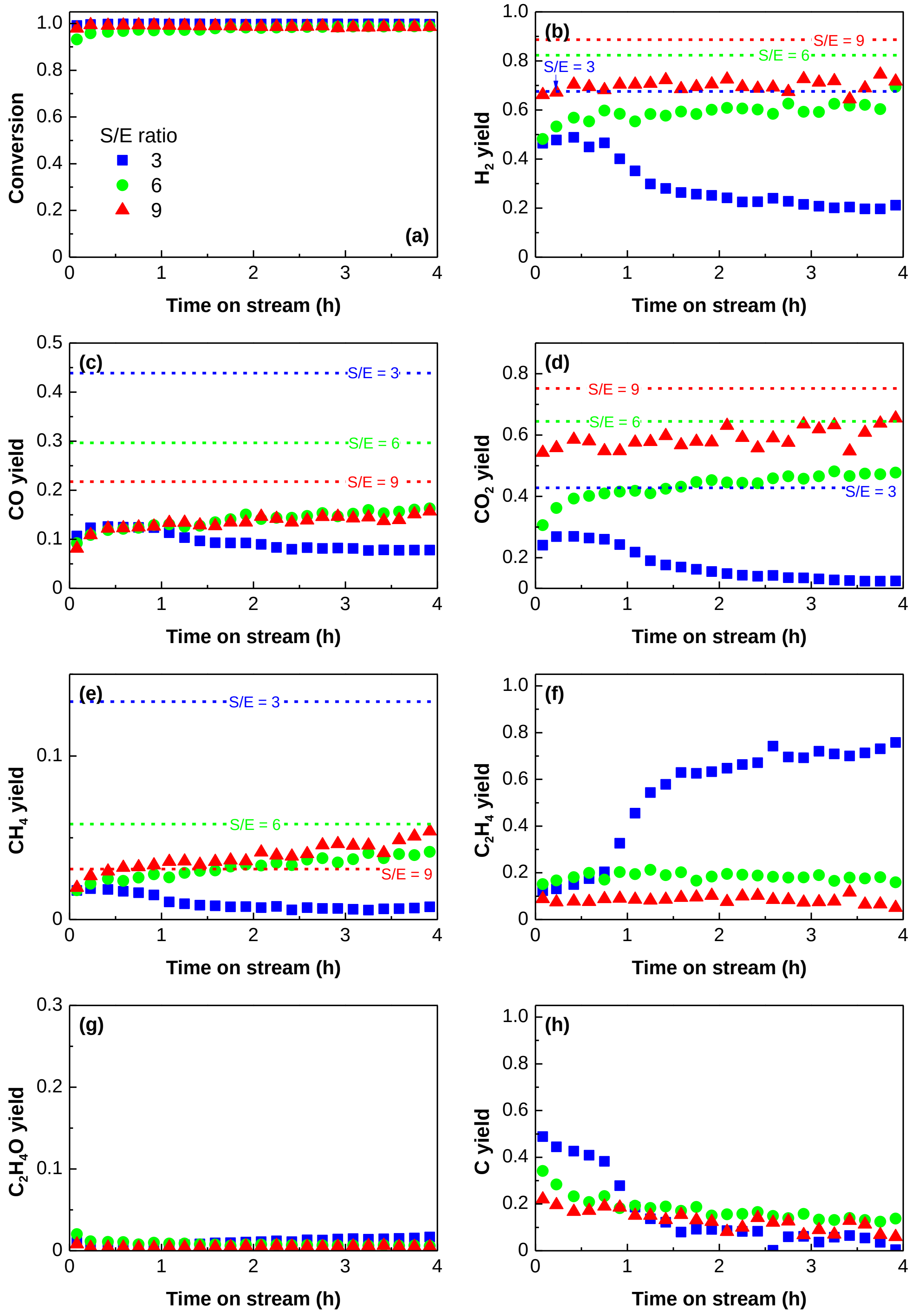


**Figure S5.** Effect of S/E ratio on the evolution of the (a) ethanol conversion and yields of (b) $H_2$, (c) CO, (d) $CO_2$, (e) $CH_4$, (f) $C_2H_4$, (g) $C_2H_4O$, and (h) carbon with time on stream. Reaction conditions: 600 ºC; space time, 0.025 h; ethanol partial pressure, 0.05 bar.

## 3. Scanning electron microscopy (SEM) analysis

The SEM images for the spent catalyst samples obtained with a S/E ratio of 3 at 500 and 650 ºC and with a space time of 0.025 h and 0.1 h are shown in Figures S6 and S7, respectively. The measurements were carried out in a Hitachi S-4800 N field emission gun scanning electron microscope using a secondary electron detector. As seen, the surface of the spent catalyst at 500 ºC (Figures S6a and S7a) is practically covered with abundant carbon filaments with heterogeneous sizes and orientations. Contrariwise, two carbon phases can be distinguished on the surface of the spent catalysts at 650 ºC (Figures S6b and S7b): an amorphous mass and carbon filaments.

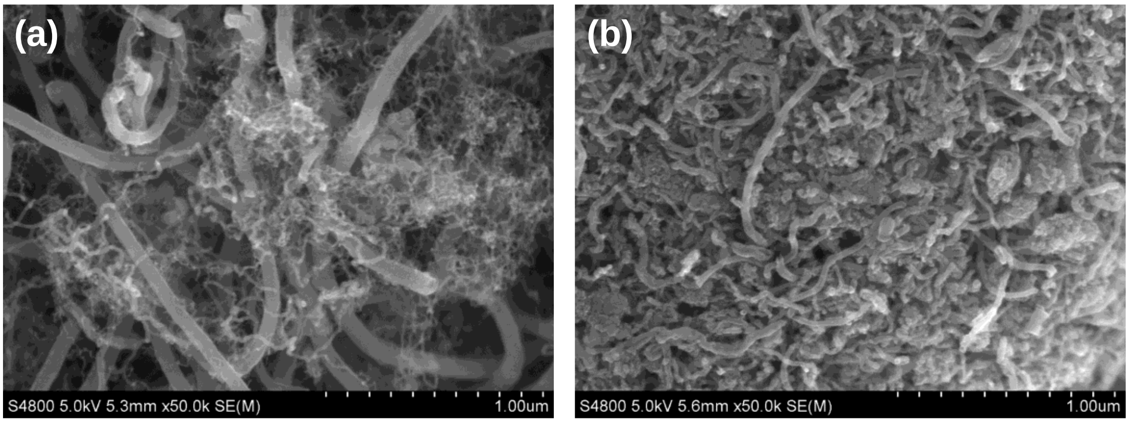


**Figure S6.** SEM images of the spent catalysts at (a) 500 ºC and (b) 650 ºC with a space time of 0.025 h.

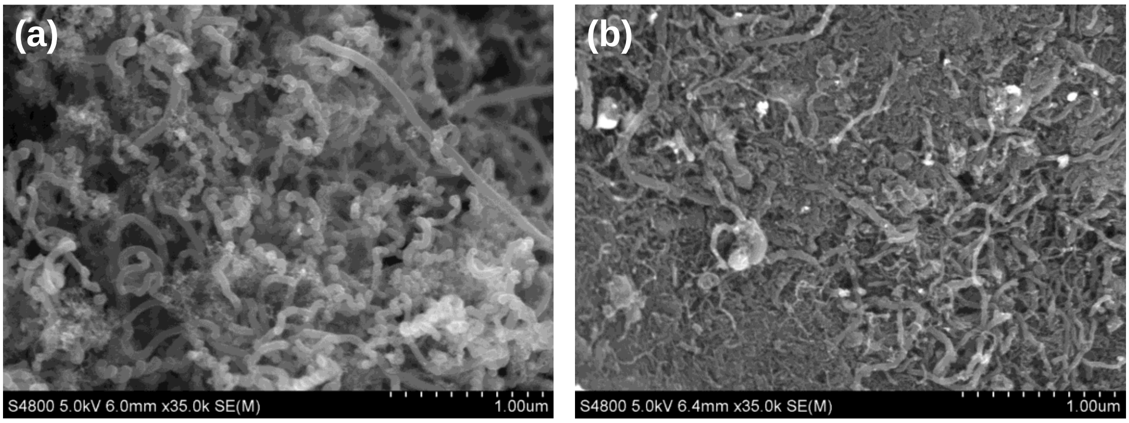


**Figure S7.** SEM images of the spent catalysts with S/E ratio of 3 at (a) 500 ºC and (b) 650 ºC with a space time of 0.1 h.
